# Validation of PTV margin for Gamma Knife Icon frameless treatment using a PseudoPatient® Prime anthropomorphic phantom

**DOI:** 10.1002/acm2.12997

**Published:** 2020-08-12

**Authors:** Eun Young Han, Parmeswaran Diagaradjane, Dershan Luo, Yao Ding, Georgios Kalaitzakis, Emmanouil Zoros, Kyveli Zourari, Themistoklis Boursianis, Evangelos Pappas, Zhifei Wen, Jihong Wang, Tina Marie Briere

**Affiliations:** ^1^ Department of Radiation Physics The University of Texas MD Anderson Cancer Center Houston TX USA; ^2^ Department of Medical Physics University of Crete Heraklion Greece; ^3^ Medical Physics Laboratory Medical School National and Kapodistrian University of Athens Athens Greece; ^4^ Department of Biomedical Sciences Radiology & Radiotherapy Sector University of West Attica Athens Greece

**Keywords:** anthropomorphic phantom, Gamma Knife Icon, gel, PTV

## Abstract

The Gamma Knife Icon allows the treatment of brain tumors mask‐based single‐fraction or fractionated treatment schemes. In clinic, uniform axial expansion of 1 mm around the gross tumor volume (GTV) and a 1.5 mm expansion in the superior and inferior directions are used to generate the planning target volume (PTV). The purpose of the study was to validate this margin scheme with two clinical scenarios: (a) the patient’s head remaining right below the high‐definition motion management (HDMM) threshold, and (b) frequent treatment interruptions followed by plan adaptation induced by large pitch head motion. A remote‐controlled head assembly was used to control the motion of a PseudoPatient® Prime head phantom; for dosimetric evaluations, an ionization chamber, EBT3 films, and polymer gels were used. These measurements were compared with those from the Gamma Knife plan. For the absolute dose measurements using an ionization chamber, the percentage differences for both targets were less than 3.0% for all scenarios, which was within the expected tolerance. For the film measurements, the two‐dimensional (2D) gamma index with a 2%/2 mm criterion showed the passing rates of ≥87% in all scenarios except the scenario 1. The results of Gel measurements showed that GTV (D_100_) was covered by the prescription dose and PTV (D_95_) was well above the planned dose by up to 5.6% and the largest geometric PTV offset was 0.8 mm for all scenarios. In conclusion, the current margin scheme with HDMM setting is adequate for a typical patient’s intrafractional motion.

## 1 INTRODUCTION

Gamma Knife radiosurgery has a long history as a treatment of choice for lesions and surgical cavities arising from metastatic disease to the brain.^1^, ^2^ The single‐fraction frame‐based treatment regimen poses a challenge in treating tumors ≥ 4 cm in maximum dimension due to the substantial risk of normal tissue toxicity such as radionecrosis.^3^, ^4^ The advent of the Gamma Knife Icon treatment system with integrated cone beam computed tomography (CBCT) and a high‐definition motion management (HDMM) system has extended Gamma Knife treatment options to single‐ or multi‐fractional treatment regimens^5^ using frameless mask‐based immobilization, and it may be used for large tumor volumes and cases involving a postoperative surgical cavity.^6^, ^7^, ^8^ The inherent ability of the integrated CBCT system to localize the acquired image in stereotactic space enables rigid co‐registration with planning images [magnetic resonance imaging (MRI) or CT], thereby eliminating the need for a stereotactic head frame. The HDMM system monitors a patient's intrafractional motion, in which an infrared marker placed on the patient's nose is used as an external surrogate to track the intracranial target motion.^8^ In clinic, a planning target volume (PTV) margin is applied to account for intrafractional motion, and the HDMM is set to an alert threshold of 1.5 mm.^8^, ^9^ To generate a PTV, uniform axial expansion of 1 mm around the gross target volume (GTV), and a 1.5 mm expansion in the superior and inferior directions were used. This margin recipe is based on the assumption that the mechanical accuracy of the mask‐based Gamma Knife delivery systems have uncertainties comparable with linac‐based treatment. The increased margin in the superior‐inferior direction accounts for greater rotational motion uncertainty about the lateral axis (X coordinates in LGK) and uncertainty from the slice thickness in the same direction. The planning goal is to achieve a coverage of 100% of the GTV volume (D_100_) and more than 95% of the PTV volume (D_95_) by the prescription dose, which is similar to linac‐based brain radiosurgery.

Although intracranial target motion is expected to be lower than for the external surrogate, studies have shown that with targets located superiorly and farther from the pivot point of head movement (close to the ears), the target motion caused by a patient’s intrafractional motion could exceed that of the external surrogate.^9^, ^10^ Hence, careful evaluation of the target by a patient's intrafractional motion is required to validate the PTV margins. Volumetric evaluation allows an assessment of the target coverage through a measured cumulative dose volume histogram (DVH).^11^ However, there have been limited studies focused on the volumetric evaluation of the dose distribution with Gamma Knife Icon frameless treatments and the impact of patient motion.

The purpose of the current study was to validate the PTV margin scheme for two clinical scenarios: the patient’s head remains below but close to the HDMM tolerance threshold (1.5 mm), and frequent multiple treatment interruptions induced by a large pitch head motion (X coordinates in LGK) followed by plan adaptation. To mimic these clinical scenarios, a remote‐controlled pitch‐adjustable head assembly^12^ was used to simulate a patient’s intrafractional motion. For dosimetric evaluation, an ionization chamber (point dose measurements), EBT3 film [two‐dimensional (2D) measurements], and polymer gel [three‐dimensional (3D) measurements] were used. These measurements were then compared with the planned doses.

## 2 MATERIALS AND METHODS

### 2.A Head phantom

A PseudoPatient® Prime head phantom (RTsafe, Athens, Greece) was used, which a 3D‐printed anatomical replica is created using the CT image of a human head. The hollow phantom with internal anatomical bony structures can be filled with water and has different inserts to hold (a) an ionization chamber for point dose measurements, (b) films for sagittal or coronal 2D plane dosimetric evaluation, and (c) a cylindrical gel insert for 3D dosimetric evaluation. The ionization chamber insert is a polymethyl methacrylate (PMMA) plug of 2 mm thick and 120 mm long and is manufactured to fit the ionization chamber. The film cassette is made of solid water with rectangular dimensions of 70 mm width and 145 mm length, and it has four metal pins for registration purposes. The gel insert consists of PMMA cylinder with dimensions of 140 mm length and 74 mm diameter.

**Fig. 1:**
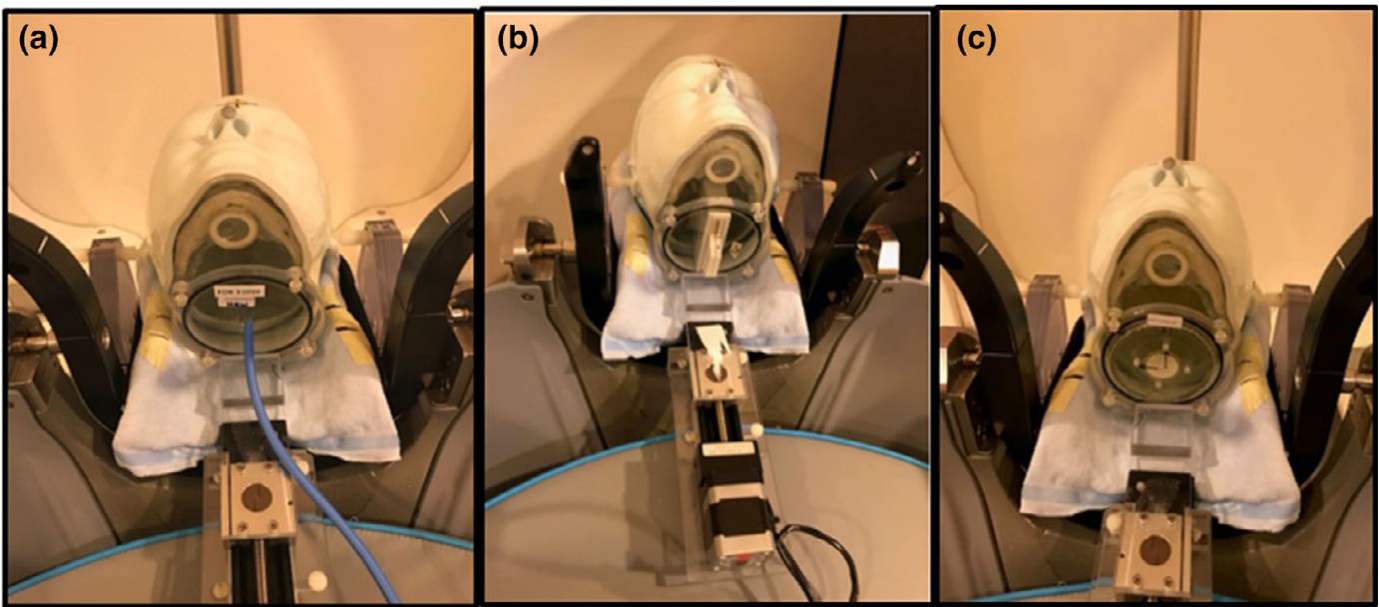
RTsafe phantom positioned in the remote‐controlled translation assembly. (a) Chamber, (b) Film, (c) Gel.

The head phantom was aligned in a neutral position using two lateral rods secured to the Gamma Knife Icon head holder; these rods can be pivoted at both ears of the phantom. Pitch rotation was accomplished by pushing the head phantom by the linear slider that was part of a translation assembly with a stepper motor (XSlide, Velmex, Bloomfield, NY) that was remotely controlled from the console area (Fig. 1).^12^

### 2.B CT imaging

Prior to treatment planning, the head phantom was positioned in the Gamma Knife Icon head holder and immobilized using a Moldcare head cushion (Alcare, Tokyo, Japan). CT images of the immobilized head phantom along with the Gamma Knife Icon head holder and Moldcare cushion were acquired using a Phillips Brilliance BigBore (Phillips, Amsterdam, Netherlands) scanner with a slice thickness of 1 mm. Three CT images of the water‐filled head phantom with the ionization chamber, film, and without inserts were acquired as reference images for point dose, 2D, and 3D dosimetric planning.

### 2.C Gamma Knife Icon treatment planning

**Fig. 2:**
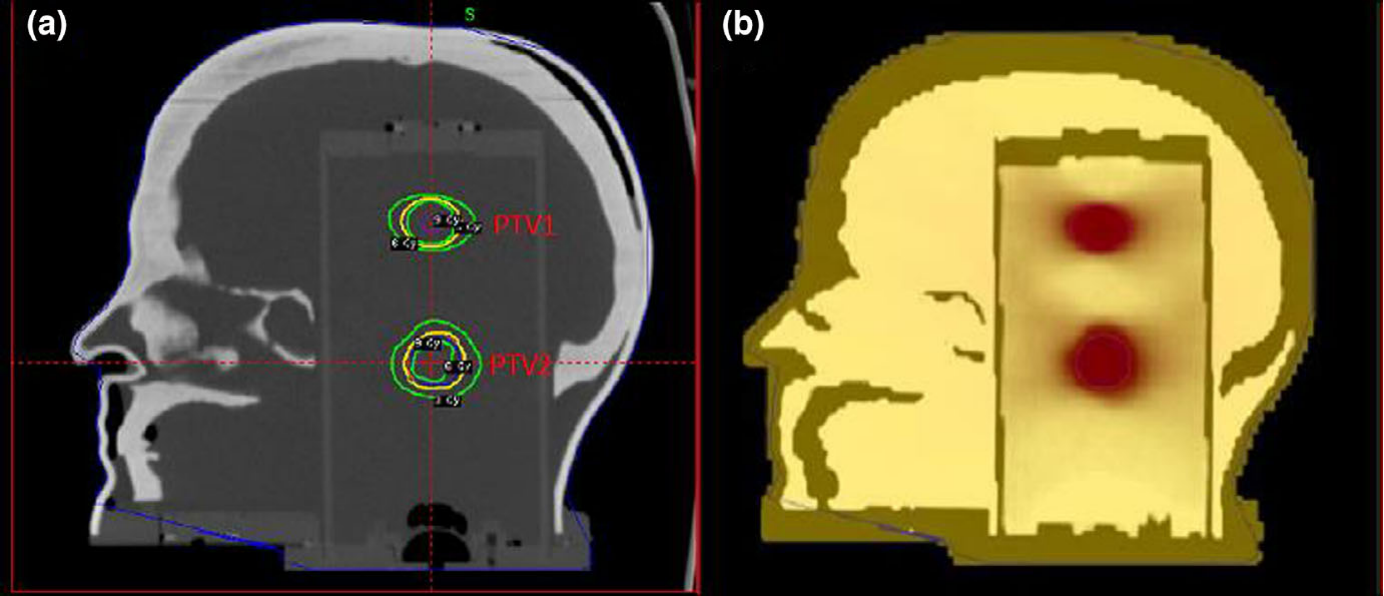
Sagittal view of the RTsafe phantom: (a) locations and planned dose distributions of PTV1 and PTV2 on the computed tomography images, (b) blended image of the image registration between post‐irradiation magnetic resonnace imaging and planned RTDose data with structures of the Gel phantom.

Leksell Gamma Plan (Ver 11.1.1; Elekta AB, Stockholm, Sweden) was used for treatment planning. Two targets (PTV1 and PTV2) were defined within the intracranial space of the head phantom. Figure 2(a) shows the locations and planned dose distributions of PTV1 (superior target) and PTV2 (inferior target). PTV1 was defined by placing a single 16 mm shot at the center of a sensitive volume of the ionization chamber, and contours were drawn to encompass the dose volume covered by the prescription isodose line (IDL). Because the gel shows a linear dose‐response up to 12 Gy,^13^, ^14^ a prescription dose of 6 Gy to the PTV was used (6 Gy to the 50% IDL). The long axis of the elliptical‐shaped PTV1 was 22 mm and the short axis was 16 mm, with a target volume of 3.98 cm^3^.

PTV2 was defined by creating a GTV2 with an 18 mm diameter and 2.99 cm^3^ volume located 5 cm inferior to the center of PTV1. Margin expansions of 1 mm in the anterior‐posterior and lateral directions and 1.5 mm in the superior‐inferior direction were applied to arrive at the final PTV2 volume of 4.32 cm^3^. The volume expansion of GTV2 was validated by using RayStation (RaySearch, Stockholm, Sweden) and the percentage volume difference between the two planning systems (RayStation vs Gamma Plan) was <1.4%. The prescription dose for PTV2 was again set to 6 Gy to the 50% IDL. Composite shots were used to cover PTV2 to create a dosimetrically realistic plan. The sizes and locations of both PTVs were limited to the physical dimension of each dosimeter insert (ionization chamber, film, and gel).

The convolution algorithm with a dose grid size of 1 mm^3^ was chosen for the absolute dose measurement comparison to better include the tissue heterogeneities within the skull and head phantom assembly. The CT image of the water‐filled phantom without inserts was used to map CT versus density for treatment planning. A total beam‐on time of 14.9 min was used.

### 2.D Simulated clinical scenarios

To evaluate the PTV margin for typical frameless treatment scenarios related to the patient's intrafractional motion, one reference scenario and two clinical scenarios were simulated. The details are as follows.

#### 2.D.1 Reference scenario

Ideal scenario without intrafractional motion or interruption of treatment.

#### 2.D.2 Treatment scenario 1

##### 2.D.2.1 Head displacement right below the HDMM threshold level but without plan adaptation

A reference CBCT (CBCT_ref_) was acquired and co‐registered to the planning CT prior to the initiation of treatment. After the source reached the position of the first shot, the head phantom with each insert was rotated to a level just below the 1.5 mm HDMM alert threshold (equivalent to a pitch head rotation by 0.8°). The treatment was not interrupted and the phantom head remained in this position during the entire treatment, without reverting back to the initial position.

#### 2.D.3 Treatment scenario 2

##### 2.D.3.1 Head displacement beyond the threshold requiring multiple treatment interruptions followed by plan adaptation

With a preset HDMM threshold of 1.5 mm, a total of three interruptions by a 5° chin‐down motion were simulated every 3.7 min using remote‐controlled pitch motions. After each interruption, a CBCT image was re‐acquired and co‐registered to CBCT_ref_. The adapted plan was then reviewed and the treatment continued until the next interruption. The simulated pitch angles were chosen following retrospective analysis of patients (n = 50) who underwent frameless Gamma Knife therapy at the institution, demonstrating an average pitch angle of 0.3° ± 1.1° and a maximum of 4.5°.

### 2.E Dosimetric measurements

The phantom was irradiated after CBCT for the phantom initial setup and plan adaptation. Because each CBCT scan contributed no more than 2.5 mGy to the total dose, CBCT doses were not subtracted from the measurements.

#### 2.E.1 Ionization chamber

The purpose of the ionization chamber measurements was to evaluate the current PTV margin scheme with the absolute dose measurements depending on the clinical scenario. The measurements were acquired with PTW 31010 Semiflex ionization chambers (PTW‐Freiburg GmbH, Freiburg, Germany; sensitive volume: 0.125 cm^3^). The dose to the chamber was measured and compared with the average dose to the contoured sensitive volume of the chamber. The sensitive volume of the chamber could be visualized in the CBCT images of the head phantom, which permitted more accurate alignment with the planning images.

In order to evaluate the dose calculation accuracy within Gamma Plan, the plans were generated using both TMR 10 and convolution algorithm by placing a single 16 mm shot with 2 Gy at 50% IDL at the center of the sensitive volume of the ionization chamber. The two planned doses were compared to the ionization chamber measurements.

#### 2.E.2 Film

The purpose of the film measurement was to evaluate the current PTV margin scheme by comparing the planar dose distribution to the plans for the clinical scenarios. EBT3 GAFchromic film (Ashland Inc., Wayne, NJ) sandwiched between film slabs was positioned in the sagittal plane of the head phantom prior to each irradiation. Analysis of the dose profiles and gamma index between the calculated and measured dose distributions were performed by RTsafe. The film was calibrated using a single‐channel protocol with the red color channel.^15^ The irradiated films were digitized with an EPSON flat‐bed color scanner (Perfection V850 Pro, Nagano, Japan) using the scanning parameters described by Makris et al.^16^ After the film scans, the net optical density values were converted into the absolute dose values. The spatial resolution of the film was 0.169 mm. The gamma index criteria (dose difference/distance to agreement [DTA]) of 2%/1 mm, 2%/2 mm, 2%/3 mm, and 10% low‐dose threshold were used to evaluate the correlation between the treatment planning system (TPS) dose and film measurement.

#### 2.E.3 Polymer gel

The purpose of the gel measurements was to evaluate the current PTV margin scheme by comparing the 3D dose distributions and dose–volume histograms (DVH) to the plans depending on the clinical scenarios. In this study, vinylpyrrolidone‐based (VIP) polymer gels were used and a detailed description of the VIP formulation and manufacturing process can be found in the literature.^13^, ^14^, ^17^

Immediately after irradiation, the polymer gels were equilibrated to the MRI scanner room temperature and MRI scans were acquired 24 h after irradiation. MRI gel scans were performed on a 1.5 T Siemens MAGNETOM Aera MRI system (Siemens Healthcare, Erlangen, Germany) using a 20‐channel head‐and‐neck phased‐array coil and the same setup devices as those employed for irradiation. T2 image scans were acquired using a half‐Fourier single‐shot turbo spin‐echo (HASTE) sequence with the following acquisition parameters: repetition time = 4230 ms, echo time = 40, 353, 931, 1380 ms, pixel size = 1.4 × 1.4 mm^2^, slice thickness = 2.0 mm. R2 maps (R2 = 1/T2), which are linearly related to the radiation dose (Gy), were post‐processed and converted to 3D dose distributions by RTsafe.^11^, ^18^

The gel measurements were normalized to the mean R2 value in a central homogeneous area (4 mm radius circle) within each PTV at the sagittal plane, and the same normalization was applied to the TPS‐calculated dose distributions. Quantitative comparison of the DVH, geometric offset and 3D gamma index were performed with the measured and calculated datasets.^19^, ^20^ The gamma index criteria were 2%/1 mm and 2%/2 mm with a 10% low‐dose threshold.

## 3 RESULTS

### 3.A Ionization chamber measurements

#### 3.A.1 Dose calculation between TMR 10 and convolution algorithm

The planned dose for a single 16 mm shot was compared with the ionization chamber measurements. The TMR 10 plan showed an overestimation of 5.7% compared with the ionization chamber measurements. The convolution dose calculations, which take into account the tissue inhomogeneity from the bony structures of the skull, head holder, and phantom assembly, showed closer agreement, within 1.3% of the ionization chamber measurements (Table 1). The absolute dose was calculated using the method described by AAPM Task Group 21,^21^ and the temperature‐pressure (P_TP_), polarity (P_pol_), and ionization (P_ion_) correction factors were obtained from measurements using the same head phantom. The energy‐dependent correction factor (k_q_) was estimated to be 1.001, assuming water‐equivalent homogeneity within the phantom.

**Table 1:** Comparison of doses from Gamma Knife plan algorithms with ionization chamber measurements.

Algorithm	Measurement	Plan	% difference
TMR 10	3.77 Gy	4.00 Gy	94.25%
Convolution	4.00 Gy	3.95 Gy	101.27%

On the basis of these observations, to better estimate the target doses and to account for tissue inhomogeneity within the skull, the convolution algorithm was used to generate plans for the treatment scenarios.

#### 3.A.2 Measurements of three scenarios with PTV1 and PTV2

The phantom was aligned using the CBCT image guidance and irradiated according to each treatment scenario, including PTV1 and PTV2. The dose to the ionization chamber was measured and compared with the mean dose to the chamber‐sensitive volume in the treatment plan (Table 2). The calculated percentage difference for both targets between the measured and planned doses was within 3% for all treatment scenarios.

**Table 2:** Comparison of calculated and measured doses (ionization chamber) for different treatment scenarios.

Convolution planned dose	Ionization chamber measurements					
	Reference		Scenario 1		Scenario 2	
	Dose	% difference	Dose	% difference	Dose	% difference
11.9 Gy	12.1 Gy	2.3	12.1 Gy	2.3	12.2 Gy	2.8

### 3.B Film measurements

#### 3.B.1 2D dose distribution and dose profile comparison

**Fig. 3:**
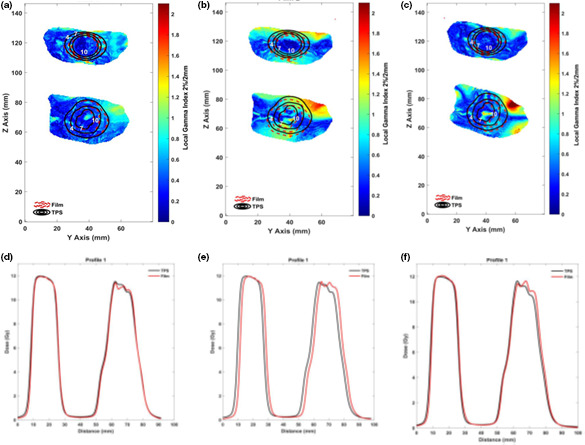
Film measured (dashed red line) and treatment planning system‐calculated (solid black line) isodose lines (Gy) superimposed on the gamma value map using passing criteria of 2%/2 mm with a threshold of 1.2 Gy (10% of the maximum dose). (a) reference scenario, (b) scenario 1, and (c) scenario 2, as well as their corresponding sagittal dose profiles (d–f).

Figure 3 shows the measured (dashed red line) and TPS‐calculated (solid black line) IDLs (Gy) superimposed on the gamma value map using the passing criteria of 2%/2 mm with a threshold of 1.2 Gy (10% of the maximum dose) as well as the corresponding sagittal profiles, for different treatment scenarios. The 2D dose distributions and sagittal dose profiles are closely aligned in the reference scenario [Figs. 3(a) and 3(d)] and in scenario 2 [Figs. 3(c) and 3(f)], where plan adaptation was implemented after each interruption. In contrast, scenario 1 [Figs. 3(b) and 3(e)] shows clear deviation between the plan and the film, which is attributable to the phantom movement equivalent to the HDMM threshold of 1.5 mm throughout the entire treatment, even though the values of the full width at half maximum of two profiles matched.

#### 3.B.2 2D gamma index comparison

2D gamma index analysis was performed using the 2%/1 mm, 2%/2 mm, 2%/3 mm criteria with a 10% low‐dose threshold (Table 3). The results showed a dramatically reduced passing rate at 2%/1 mm in all scenarios. This is attributed to the uncertainty of the film dosimetry protocol, as Makris et al. reported the film‐to‐CT image registration uncertainty to be in the order of 1.5 mm.^16^, ^22^

**Table 3:** Film two‐dimensional (2D) gamma index, comparing with the treatment planning system (TPS)‐calculated dose distributions using 2%/1 mm, 2%/2 mm, and 2%/3 mm passing criteria with 10% low‐dose threshold.

	Passing criteria	Reference	Scenario 1	Scenario 2
**PTV1**	2%/1 mm	74.5	45.5	79.5
	2%/2 mm	98.3	87.8	99.1
	2%/3 mm	100.0	98.7	99.9
**PTV2**	2%/1 mm	78.1	42.6	56.5
	2%/2 mm	98.9	75.2	87.1
	2%/3 mm	100.0	96.6	95.7

PTV, planning target volume.

The gamma passing rates with the 2%/2 mm criteria for the reference scenario were 98.3% and 98.9% for PTV1 and PTV2, respectively. In contrast, the passing rates for scenario 1 were 87.8% and 75.2% for PTV1 and PTV2. This is due to the prominent shift by 1.5 mm HDMM observed in the profile measurement [Fig. 3(e)] in addition to the aforementioned film‐to‐CT image registration uncertainty in the film dosimetry. Lastly, the gamma passing rates with the 2%/2 mm criteria for scenario 2 were 99.1% and 87.1% for PTV1 and PTV2. The gamma passing rates with the 2%/3 mm criteria for all scenarios were 95.0% or higher.

### 3.C Polymer gel measurements

Image registration between the post‐irradiation MRI and planned TPS data using the structures within the gel phantom was performed to align each target to its planned location. The geometric accuracy of 3D gel dosimetry is in the order of 1 mm since the final dose grid derived from the gel measurements after post‐imaging analysis has a resolution of 1 mm^3^. Figure 2(b) shows image registration of post‐irradiation MRI images and planned TPS data. This is to demonstrate the coincidence of each treated target to its planned location.

#### 3.C.1 DVH comparison

Comparison between the planned and measured relative dose distributions was presented in terms of cumulative DVHs for both PTV1, GTV2 and PTV2. The dose distributions were normalized to the corresponding D_50_ metric (the dose received by at least 50% of the volume) for each target. The measured D_95_ values of PTVs were higher than the planned values by up to 5.6% for all three scenarios. The measured D_100_ values of GTV2 were also higher by up to 2.6% than the planned value in all scenarios (Table 4).

**Table 4:** Comparison of gel‐measured and calculated dose–volume metrics, D_95_ for different treatment scenarios.

Target	Estimated D_95_ or D_100_ values						
	TPS	Reference		Scenario 1		Scenario 2	
		Measured	Difference	Measured	Difference	Measured	Difference
PTV1 (D_95_)	62.0%	67.6%	5.6%	66.6%	4.7%	65.1%	3.1%
PTV2 (D_95_)	76.3%	78.1%	1.9%	76.2%	0.0%	77.5%	1.3%
GTV2 (D_100_)	70.5%	73.1%	2.6%	70.5%	0.0%	72.1%	1.6%

PTV, planning target volume; TPS, treatment‐planning system.

#### 3.C.2 Geometric offset

Center‐to‐center offsets were measured independently for each target by comparing the difference in the 3D center‐of‐mass of each target between the gels (polymerized area) and plans (high‐dose area). The center‐of‐mass was calculated by averaging the distributions of the centers‐of‐mass derived by various ranges of dose thresholds, taking into account the dose gradient of each target.^17^, ^23^ Table 5 shows the largest geometric offset (0.8 mm) caused by 1.5 mm HDMM head rotation without plan adaptation in scenario 1.

**Table 5:** Geometric offset between the centers‐of‐mass of planned and measured (Gel) dose distributions for PTV1 and PTV2.

Target	Reference	Scenario 1	Scenario 2
PTV1	0.2 mm	0.8 mm	0.2 mm
PTV2	0.5 mm	0.8 mm	0.2 mm

PTV, planning target volume.

#### 3.C.3 3D gamma index comparison

Gamma index calculations were performed in 3D using passing criteria of 2%/1 mm and 2%/2 mm with a low‐dose cutoff threshold of 10%. For the defined targets, gamma index comparison was performed within a volume of interest encompassing PTV1 and PTV2. The gamma passing rates with the 2%/1 mm criteria for the scenario 2 were 96.7% and 88.6% for PTV1 and PTV2, respectively. The 3D gamma passing rate with 2%/2 mm criteria for all three scenarios was >98%, as shown in Table 6.

**Table 6:** Three‐dimensional (3D) gamma index, comparing gel‐measured with the treatment‐planning system (TPS)‐calculated dose distributions using 2%/1 mm and 2%/2 mm passing criteria with 10% low‐dose threshold.

	References	Scenario 1	Scenario 2
PTV1			
2%/1 mm	97.8	94.4	96.7
2%/2 mm	99.6	99.2	99.3
PTV2			
2%/1 mm	99.8	96.4	88.6
2%/2 mm	100.0	100.0	98.4

PTV, planning target volume.

## 4 DISCUSSION

Validation of the PTV margins for GK Icon frameless treatments via end‐to‐end testing is critical to assure target coverage and protection of organs at risk due to a patient's intrafractional motion. The purpose of the current study was to validate the PTV margin scheme using three different dosimeters for one reference scenario and two clinical scenarios: (a) the patient's head remains below but close to the 1.5 mm HDMM tolerance threshold, and (b) frequent treatment interruptions induced by large pitch head motion followed by plan adaptation. An anthropomorphic head phantom was used to simulate the treatment scenarios.

For the dose calculations, the convolution algorithm was used instead of TMR 10 because the TMR 10 plan showed a dose overestimation of 5.7% compared with the ionization chamber measurements. The dose differences between the TMR 10 and convolution algorithms correlate well with other reported studies. Rojas‐Villabona et al. reported that dose calculations generated by the convolution algorithm closely matched the measurement values with an average treatment time that was 5.9% longer than in the TMR 10 algorithm.^24^ Choi et al. reported that Monte Carlo dose calculations were lower than those calculated by TMR 10 by about 4%.^25^

The percentage difference between the ionization chamber‐measured and planned doses using the convolution algorithm were within 3%, which is the anticipated overall measurement uncertainty due to source calibration, measurement setup, and the use of an inhomogeneous phantom instead of a water phantom for the absolute dosimetry.

For the film analysis with 2%/2 mm criteria and the gel analysis with 2%/1 mm criteria, the lower passing rate by 8–12% for PTV2 over PTV1 in scenario 2 might be attributable to the larger instability of composite shots (PTV2) by plan adaptation due to the positional deviation than that with a single shot (PTV1). Luo et al. reported that plans consisting of non‐composite shots showed greater dosimetric robustness by the plan adaptation to positional deviations such as a patient’s intrafractional motion compared to the plans using composite shots.^26^

The results of volumetric dose analysis with gel measurements to assess the target coverage showed the following: first, it verified that the adequacy of the PTV margin scheme with an HDMM threshold of 1.5 mm by showing the full dose coverage for GTV2 and a higher D_95_ for both PTVs than the plans regardless of simulated treatment scenarios and second, the largest geometric offset of 0.8 mm demonstrated the intracranial target motion is again lower than the motion of the nose marker (1.5 mm HDMM).

However, the 2D film and 3D gel gamma passing rates cannot be directly compared since the results of the film dose measurements were directly compared to the planned absolute values (in Gy) in a single plane while the gel dose measurements were normalized to the relative measured doses (in %) over the entire 3D volume. And the result of this study might be limited to the specific sizes and locations of the PTVs and simulated treatment scenarios.

In summary, three different dosimeters were used to validate the PTV margin for Gamma Knife Icon frameless treatments. For the absolute dose measurement using an ionization chamber, the percentage differences for both targets were <3.0% for all scenarios, which was within the expected tolerance. For the film measurements, the 2D gamma index with the criteria of 2%/2 mm showed the passing rates of ≥87% in all scenarios except the scenario 1. Gel inserts were used for 3D volumetric evaluation as an end‐to‐end test. The results showed sufficient dose coverage on GTV/PTVs in all clinical scenarios.

## 5 CONCLUSIONS

Three different measurement methods (ionization chamber, film, and gel) were used with an anthropomorphic head phantom to validate the PTV margin scheme for Gamma Knife Icon frameless treatments. Compared to two other dosimeters, the 3D volumetric analysis with the gel dosimeter was able to show the adequate GTV/PTV coverage in various clinical scenarios, therefore, the current margin scheme with HDMM setting is sufficient for a typical patient’s intrafractional motion.

## CONFLICT OF INTEREST

The authors declare no conflict of interest.
